# Identification of a gene associated with avian migratory behaviour

**DOI:** 10.1098/rspb.2010.2567

**Published:** 2011-02-16

**Authors:** Jakob C. Mueller, Francisco Pulido, Bart Kempenaers

**Affiliations:** 1 Department of Behavioural Ecology and Evolutionary Genetics, Max Planck Institute for Ornithology, 82319 Starnberg, Germany; 2 Departamento de Zoología y Antropología Física, Universidad Complutense de Madrid, 28040 Madrid, Spain

**Keywords:** *ADCYAP1*, PACAP, migratory restlessness, bird migration, neuropeptide

## Abstract

Bird migration is one of the most spectacular and best-studied phenomena in behavioural biology. Yet, while the patterns of variation in migratory behaviour and its ecological causes have been intensively studied, its genetic, physiological and neurological control remains poorly understood. The lack of knowledge of the molecular basis of migration is currently not only limiting our insight into the proximate control of migration, but also into its evolution. We investigated polymorphisms in the exons of six candidate genes for key behavioural traits potentially linked to migration, which had previously been identified in several bird species, and eight control loci in 14 populations of blackcaps (*Sylvia atricapilla*), representing the whole range of geographical variation in migration patterns found in this species, with the aim of identifying genes controlling variation in migration. We found a consistent association between a microsatellite polymorphism and migratory behaviour only at one candidate locus: the *ADCYAP1* gene. This polymorphism explained about 2.6 per cent of the variation in migratory tendency among populations, and 2.7–3.5% of variation in migratory restlessness among individuals within two independent populations. In all tests, longer alleles were associated with higher migratory activity. The consistency of results among different populations and levels of analysis suggests that *ADCYAP1* is one of the genes controlling the expression of migratory behaviour. Moreover, the multiple described functions of the gene product indicate that this gene might act at multiple levels modifying the shift between migratory and non-migratory states.

## 1. Introduction

Each year roughly 50 billion birds, involving about half of all avian species, perform some type of migratory movement [1]. This behaviour has fascinated lay people and scientists alike, and is probably one of the biological phenomenon with the longest research tradition [1,2]. Despite extensive research over decades, the molecular, physiological and endocrinological mechanisms underlying the regulation of migratory movements remain largely unknown [2–6]. In particular, it remains unclear whether endocrine changes are a cause or an effect of migratory processes [3]. There is a clear need to study the molecular genetic basis of migration, which may bridge the gap between genes and phenotype. Knowledge of genetic differences and differential gene expression between migratory and non-migratory individuals will help in solving the causality problem encountered in purely physiological studies. Studies on the molecular basis of migration may also help to improve our understanding of the evolutionary history involved in changes in migratory behaviour in response to past environmental shifts.

Before and during migration, migratory birds undergo a profound and synchronized shift in a set of physiological adaptations and behavioural traits. Migratory disposition, i.e. a state of readiness for prolonged flights, comprises hypertrophy of flight muscles, fat deposition in the adipose tissue, integrated changes in enzyme activities involved in the energy metabolism, hyperphagia, dietary changes and the development of migratory activity [4,7]. From a behavioural ecology perspective, the ability to shift circadian activity during migration represents a key element of avian migratory behaviour. Indeed, many species of diurnally active birds switch to additional nocturnal activity during the migratory season [4]. Such a change in the circadian activity patterns involves substantial physiological and hormonal shifts. Birds migrating at night maintain high levels of physical and cognitive functions such as prolonged flight, navigation performance and alertness against predators at times when they usually sleep [8]. It is suggested that components of the endogenous circadian clock control the nocturnal migratory behaviour [9–12]. Personality traits have also been discussed in the context of variation in migratory behaviour. For example, it has been suggested that initiation of migration behaviour and migration distance are related to individual competitive ability or dominance [13], which in turn may be linked to aggression and anxiety-related behaviour [14]. Furthermore, migratory and non-migratory birds may differ in exploratory behaviour [15–17].

Many components of migratory behaviour, such as the amount, timing and intensity of migratory activity, are under strong genetic control, at least in small night-migrating passerines [18–21]. Also cross-breeding experiments among groups of European blackcaps (*Sylvia atricapilla*) that differed in migratory behaviour indicated a strong genetic basis of this behaviour [22,23]. In the European blackcap, a model species for the study of avian migration, a new wintering area was established in only a few decades, involving evolutionary changes in migration distance and direction [24,25]. Moreover, a strong evolutionary reduction of migratory activity has been observed in a blackcap population, presumably in response to climate change [26]. These results suggest that the evolution of migratory behaviour in a resident population or of residency in a migratory population may be a common and rapid process [21]. Furthermore, quantitative genetic studies provide evidence for the genetic integration of migratory traits [20,27]. High genetic correlations among incidence, amount, intensity and timing of migratory activity in blackcaps suggest that these components of migratory behaviour are influenced by common genetic mechanisms [20,27]. As a consequence, we would expect that phenotypic variation of correlated migratory traits is linked to genetic variation at a single closely linked gene cluster or a few ‘regulatory genes’ with multiple pleiotropic effects [28].

Based on two behavioural elements of avian migration, nocturnality and exploratory behaviour, we selected nine exonic polymorphic loci in six candidate genes. The loci are reported microsatellites and single-nucleotide polymorphisms (SNPs) in the exons (coding and untranslated exonic regions (UTRs)) of candidate genes in birds that are known to be involved in the expression of circadian rhythms [29] or personality traits [30–32]. We made use of the profound knowledge on blackcap migration and of the large amount of geographical and within-population variation in migratory behaviour found in this species [21,33] to study the association between allelic variation at candidate loci and migratory behaviour at two levels: (i) among individuals within populations, and (ii) between populations that vary in the proportion of migrants and in migration distance. Here, we show that migratory restlessness is consistently associated with allele length at a 3′-UTR locus of the adenylate cyclase-activating polypeptide 1 (*ADCYAP1*) gene in two independent populations. Moreover, the same alleles are correlated with an estimated higher proportion of migratory individuals across 14 blackcap populations.

## 2. Material and methods

### (a) Samples

**Figure 1.**
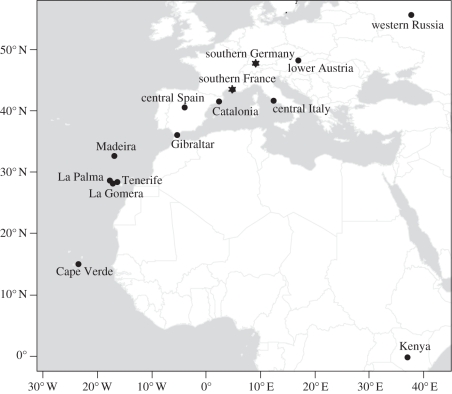
Map of the sampled blackcap populations. The two populations, which were used for the intrapopulational analyses are indicated with stars.

Thirteen European/African blackcap populations representing the entire range of geographical variation in migration patterns, from Cape Verde to western Russia, have been sampled in the years 1989–1996 (figure 1). We also included a sample of birds captured in Kenya in the year 2000. The geographical coordinates and sampling information for each population are listed in electronic supplementary material, table S1. All birds investigated were sampled randomly within a restricted geographical area, which we defined as population. Birds held in captivity (Madeira, southern France, southern Germany, western Russia) were collected as nestlings from the populations in the wild. All other birds—except those from central Italy and Kenya—were captured with mistnets in the wild during or after the reproductive season, but before the start of migration. The samples of central Italy and Kenya were captured with mistnets during winter.

Blood samples (*ca* 50 µl) were obtained by puncturing the brachial vein. An isotonic NaCl–EDTA buffer (0.85% NaCl) was used to prevent blood cell lysis and coagulation. For the genetic analyses, the erythrocyte fraction was used as a source of DNA.

### (b) Determination of migratory status of individuals and populations

Migratory behaviour of individuals from the southern France and southern Germany populations was quantified by measuring nocturnal migratory activity in registration cages under identical, standardized conditions. These measures of migratory activity have been analysed and published previously (southern France: [27,34] and southern Germany: [18,26]). The amount of migratory activity was measured in inexperienced hand-reared birds as the total number of 30 min intervals with activity during the autumn migration period. This variable is correlated to the distance the bird would migrate in the wild [4].

The migratory status of each population was classified using all available information on the migratory behaviour of individuals from that population, including capture–recapture data, direct observations and laboratory measures of migratory restlessness (Cape Verde, Tenerife, Madeira, southern France, southern Germany, lower Austria and western Russia). Integrating this information, we classified blackcaps from Cape Verde [23] and Gibraltar [35,36] as being completely resident, blackcaps from Tenerife [24], Madeira [37] and, presumably, La Gomera and La Palma as residents, but showing some residual migratory restlessness in the laboratory. Blackcaps breeding in Catalonia (G. Gargallo 1992, personal communication) and southern France [27,34] are partially migratory. Birds breeding in the uplands north of Madrid, central Spain, are migratory but, presumably, migrate only short distances to the south of the Iberian Peninsula [36]. The blackcap populations breeding in southern Germany and lower Austria and the winter sample of central Italy are presumably completely migratory with largely intermediate migration distances [38,39]. Finally, individuals from western Russia and the winter sample of Kenya are classified as distinct long-distance migrants (all birds covering distances greater than 3500 km). This pattern is in general agreement with a leap-frog migration pattern [1].

### (c) Genotyping

We selected all known exonic di- and trinucleotide microsatellite loci in candidate genes for circadian behaviour: *CLOCK, ADCYAP1, CREB1* and *NPAS2* (for selection strategy, primer, PCR and scoring details see [29]). We also genotyped one exonic trinucleotide microsatellite and four exonic SNPs of the major candidate genes for ‘exploratory behaviour’ or ‘anxiety-related behaviour’: *DRD4* and *SERT* (for details, see electronic supplementary material, table S2). This set represents a complete list of all currently known microsatellites in exons of candidate genes for circadian behaviour and personality in birds [29,31]. We focused on exonic microsatellites because they are mostly conserved across species, thus promoting primer transferability. As there was no microsatellite in the *DRD4* candidate gene, we identified blackcap-specific SNPs by sequencing eight blackcap individuals at exon 3 of the *DRD4* gene. This limited set of easy accessible polymorphisms with exonic location has been selected because of their *a priori* chance to directly influence gene product structure and gene expression.

For comparative association analyses, we used eight anonymous di- and tetranucleotide microsatellite loci (Syl1, Syl2, Syl4, Syl5, Syl6, Syl9, Ppi2 and Pca8), which are presumably neutral (for genotyping details see [40] and electronic supplementary material, table S2). All birds were sexed using Griffiths *et al*.'s [41] P2 and P8 primers. An ABI 3130 sequencer was used for the microsatellite fragment analysis and the ABI SnaPshot protocol for SNP genotyping.

### (d) Data analyses

We tested associations between individual migratory restlessness and genotypes using mixed-effects regression models. Because the samples of southern France and southern Germany comprised some sibships (i.e. nestlings collected from the same nest), we minimized the effect of pseudoreplication by including sibship as a random factor in the models (R package lme4 [42,43]). Sibship was always significant (*p* < 0.002; tested with R package RLRsim [44]) and the variance owing to sibship was estimated as 43 and 29 per cent of the total variance in southern France and southern Germany, respectively, reflecting the high heritability in this trait [18,26]. We also tested the interaction between sex and genotype, but this was not significant in both populations. This term was, therefore, excluded from the final model. All *p*-value and standardized regression coefficient estimates are based on 10 000 Markov chain Monte Carlo (MCMC) samples (R package languageR [45]). Individual microsatellite genotypes were coded as mean allele lengths averaged over the two alleles and SNP genotypes were coded according to the allele dose model (copy number of one of the two alleles) in the within-population analyses. These standard genotype coding models are powerful with one degree of freedom, and are known to capture most allelic effects in association studies [46]. Note, however, that these models implicitly assume that alleles are codominant and that allele length is linearly related to variation in the phenotype.

We analysed the relationship between the migratory status and the genetic composition of populations using partial Mantel tests with 10 000 permutations on appropriate distance matrices accounting for geographical distances ([47], R package ecodist [48]). In samples where sibships were included (i.e. Madeira, southern France, southern Germany and western Russia), we randomly selected one individual per sibship. The genetic differentiation at each locus and for all population pairs was calculated as multi-allelic *F*_ST_-values according to Weir & Cockerham [49] using Genepop [50]. Distances in migratory status were calculated after coding pure resident populations as ‘0’, resident populations with some migratory restlessness as ‘0.5’, partial migratory populations as ‘1’, completely migratory populations migrating short-distances as ‘1.5’, intermediate-distance migratory populations as ‘2’ and distinct long-distance migratory populations as ‘2.5’ (electronic supplementary material, table S1). Surface geographical distances were calculated using the R package gmt [51]. Genetic variance partitioning among groups of differing migration status was performed in a hierarchical analysis of molecular variance (AMOVA) framework with 10 000 permutations using Arlequin [52].

## 3. Results

### (a) Within-population tests

**Figure 2.**
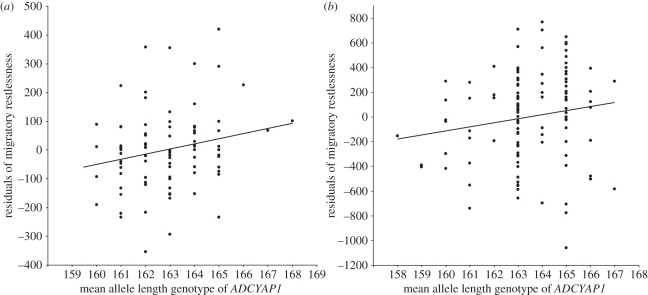
Mean allele length genotypes at the *ADCYAP1* locus plotted against residuals of the null mixed-effects model on migratory restlessness with sibship as random factor and no fixed effect (genotype). (*a*) Southern France and (*b*) southern Germany.

In both populations with data on individual migratory activity (southern France and southern Germany; figure 1), migratory restlessness was associated with the genotypes of the *ADCYAP1* locus (table 1). Individual mean allele length at *ADCYAP1* correlated positively with migratory restlessness in both populations (figure 2). The mean genotypes explained 2.7 and 3.5 per cent of the variance in migratory restlessness in southern France and southern Germany, respectively. Note that the test in the southern France population failed nominal significance (*p* = 0.056). We consider this a type II statistical error, given the smaller sample size, and the consistency of allelic effects in terms of strength and direction in both populations and in the among-population test (see below). We also explored two alternative genotype coding models at the *ADCYAP1* locus by using the shorter (or longer) allele in each individual as a measure of genotype. Whereas the ‘longer allele’ model was always non-significant (*p* > 0.22), the ‘shorter allele’ genotypes were significantly associated with migratory restlessness in both southern France (*p* = 0.048; standardized regression coefficient = 0.21) and southern Germany (*p* = 0.022; standardized regression coefficient = 0.21). This could indicate that the shorter alleles are more effective in influencing migratory restlessness in these populations than longer alleles (but see among-population results). We also found significant associations with migratory restlessness at two control microsatellite loci (table 1), but these were not consistent across the within- and among-population tests.

**Table 1.** Results (*p*-values) of three independent association tests between variation in migratory behaviour and genetic variation at 17 polymorphic loci. n.a., not applicable owing to monomorphic locus. *p*-values < 0.05 are in bold; 0.05 < *p*-value < 0.1 in bold italic.

gene/locus name	southern France^a^ (*n* = 87)^c^	southern Germany^a^ (*n* = 119)^c^	all populations^b^
*CLOCK*	0.11 (0.16)	0.76 (0.03)	0.59 (−0.04)
*ADCYAP1*	***0.056*** (0.24)	**0.025** (0.20)	**0.0038** (0.45)
*CREB1*	0.74 (−0.04)	0.12 (0.14)	0.80 (−0.15)
*NPAS2*	n.a.	0.76 (0.03)	n.a.
*SERT*	0.67 (0.05)	n.a.	n.a.
*DRD4*_366	0.34 (−0.04)	0.24 (−0.09)	0.83 (−0.17)
*DRD4*_524	0.39 (−0.02)	n.a.	n.a.
*DRD4*_815	0.59 (0.01)	n.a.	n.a.
*DRD4*_890	0.50 (−0.02)	0.91 (0.01)	0.70 (−0.08)
Syl1	0.31 (0.10)	0.91 (0.01)	0.82 (−0.15)
Syl2	**0.046** (0.22)	0.27 (−0.11)	0.11 (0.23)
Syl4	0.61 (0.05)	0.30 (0.09)	0.75 (−0.11)
Syl5	0.85 (−0.04)	0.40 (−0.07)	0.77 (−0.11)
Syl6	0.10 (0.19)	0.60 (−0.05)	0.21 (0.15)
Syl9	0.74 (0.03)	0.21 (−0.12)	0.16 (0.19)
Ppi2	**0.020** (−0.27)	***0.063*** (−0.17)	0.83 (−0.15)
Pca8	***0.088*** (−0.19)	0.70 (−0.03)	0.44 (0.004)

^a^Individual-based association between migratory restlessness and mean allele length genotype (microsatellites) or allele dose genotype (SNPs) using mixed-effects models with sibship as random factor (in brackets MCMC estimate of standardized regression coefficient).

^b^Population-based correlation between migration status distances and genetic differentiation (FSTs) among all summer population samples using a partial Mantel test conditional on geographical distances (in brackets Mantel correlation coefficient).

^c^Actual sample sizes. The smaller sample sizes in comparison to the ones presented in electronic supplementary material, table S1 are explained by the fact that migratory restlessness was not measured in all birds for which DNA samples were available.

### (b) Among-population tests

We found a strong general correlation between the between-population differentiation in migration status and genetic differentiation at the *ADCYAP1* locus, but not at any other locus (table 1). To account for geography-based genetic similarity between breeding populations owing to common colonization histories and gene flow, the analysis included surface geographical distances between sample sites, and excluded the winter samples of central Italy and Kenya, for which the breeding areas of the birds could not be determined. The results of this analysis were robust against slightly different estimates in migration status for populations with little migration data. The genotypic variance at the *ADCYAP1* locus explained 2.6 per cent of the variance among groups of populations differing in migration status (AMOVA). It is thus similar to the variance in migratory restlessness among individuals explained by this polymorphism. Note, however, that this estimate might be inflated because the AMOVA does not account for genetic similarity owing to geographical proximity of the populations.

**Figure 3.**
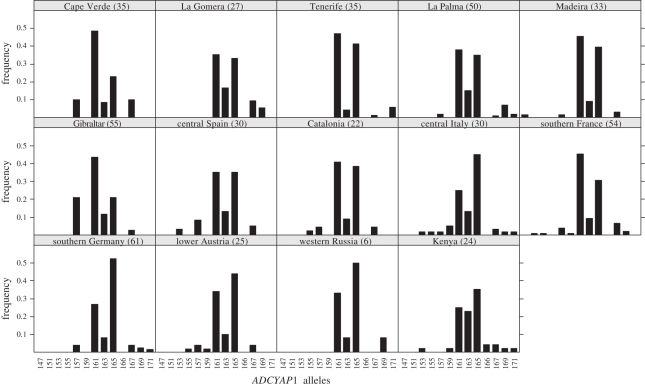
Allele frequency distributions of the *ADCYAP1* locus in 14 blackcap populations (sample sizes in brackets).

**Figure 4.**
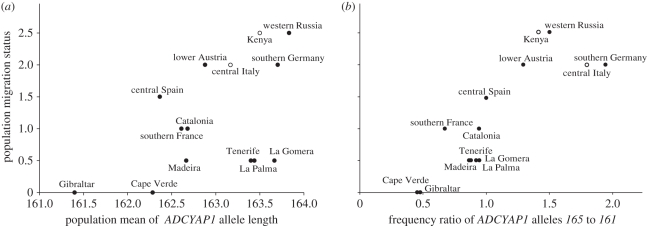
The association between population migration status and (*a*) population mean of *ADCYAP1* allele length (Spearman rank correlation: *ρ* = 0.57, *p* = 0.034) or (*b*) the frequency ratio between *ADCYAP1* alleles *165* and *161* (Spearman rank correlation: *ρ* = 0.87, *p* = 0.000054). Here, the winter samples of Kenya and central Italy are included in the analyses.

The distance approach used above does not provide information on the direction of the relationship between migration status and allele length. To explore the direction of the relationship, we directly tested the correlation between population migration status and population mean allele length and found a significant positive correlation (Spearman rank correlation: *ρ* = 0.57, *p* = 0.034; figure 4*a*). We also tested this relationship with alternative population summary statistics, such as median allele length (electronic supplementary material, figure S1), mean of the shorter (or longer) allele in each individual (electronic supplementary material, figure S1), proportion of allele *161* or shorter (Spearman rank correlation: *ρ* = −0.78, *p* = 0.0011) and proportion of allele *165* or longer (Spearman rank correlation: *ρ* = 0.62, *p* = 0.017). The allele frequency distribution at the *ADCYAP1* locus showed a bimodal pattern in all populations (figure 3). The most frequent alleles (*161* and *165*) are two mutational steps apart, assuming a stepwise mutation model, and show considerable frequency variation among populations. As this pattern is indicative of an old balanced polymorphism, we also tested the frequency ratio of these two major alleles as a potential predictor for population migration status. Migration status was significantly linked to this frequency ratio (*165* to *161*) (Spearman rank correlation: *ρ* = 0.87, *p* = 0.000054), whereby the longer allele was more prevalent in more migratory populations (figure 4*b*).

All but one of the seven—non-independent—tests of the among-population effect were significant, indicating the robustness of the association between population migration status and allele length at the *ADCYAP1* polymorphism. The combinatorial probability of finding three significant effects at the same locus in the same direction in three independent studies (two within- and one between-population study) on 14 loci each (here, the four *DRD4* loci are treated as one locus) by chance equals 0.00017.

## 4. Discussion

In this study, we investigated the effect of nine known exonic polymorphisms in six candidate genes for behavioural traits on the expression of migratory behaviour in a migratory bird species. Three independent tests indicate that long alleles at a microsatellite in the 3′-UTR of the *ADCYAP1* gene are associated with high migratory activity in blackcaps, either measured as migratory restlessness of individuals in the laboratory or assessed as the proportion of migrants and migration distance in natural populations. Both the within-population analyses and the among-population comparison indicate that genotypic variation at this gene explains about 3 per cent of phenotypic variation in ‘migratoriness’, which amounts to a maximum of 6–8% of the additive genetic variance in this trait, assuming a mean heritability of this trait of 0.43 [26]. This represents a relatively large single-gene effect on a complex behavioural trait when compared with reported genetic effects on other complex traits [53,54]. There are only few other gene variants reported to influence a behavioural trait in a wild bird population with a similar strength, for instance, an exonic *DRD4* SNP explaining about 5 per cent of the exploratory behaviour in great tits [31,32]. The large proportion of unexplained additive genetic variance indicates that many additional still unknown loci contribute to the expression of migratory behaviour. In general, complex traits are expected to show a genetic architecture with a high number of contributing genes with epistatic effects and gene by environment interactions [53,55].

The *ADCYAP1* polymorphism is located in the 3′-UTR of the gene, which is known to comprise important regulatory elements of post-transcriptional processes [56–58]. It has been suggested that the insertion of simple sequence repeats in 3′-UTR regulatory elements and the structural variation at the 3′-UTR mediated by microsatellite variation can interact with and modify the 3′-UTR regulatory functions [59,60]. The polymorphism could also be in linkage disequilibrium with a different functional polymorphism in the gene region influencing peptide structure or transcription level. The allelic association, however, needs to be consistent across the populations to explain the observed patterns. Only direct expression studies in different tissues can reveal the link between genotypes and levels of the different splice variants and/or isoforms at the *ADCYAP1* gene [61]. General functionality of the *ADCYAP1* polymorphism is indicated by conservation across avian and mammalian species. A similar dinucleotide sequence repeat in the 3′-UTR with different levels of motif purity and polymorphism (where tested) has been found in 40 bird species, human, mouse and rat ([29,62]; our unpublished data; UCSC genome browser at http://genome.ucsc.edu/). Obviously, only further work on other species will show to what extent the association between the polymorphism and the expression of avian migration can be generalized.

The *ADCYAP1* gene encodes the pituitary adenylate cyclase-activating polypeptide (PACAP), which is one of the most studied neuropeptides (more than 3000 papers deal directly with PACAP) ([61], Web of Science at http://apps.isiknowledge.com). The peptide and its receptors are widely distributed in the brain and in various peripheral organs [61,63]. In comparison with the products of our other tested candidate loci, PACAP has a broad spectrum of biological functions with profound influence on physiology and behaviour. Most of the reported effects exerted by PACAP are indeed strongly linked to the physiological and behavioural shifts described for avian migration. For example, exposure of the chicken pineal gland to PACAP induces a transitory increase in melatonin secretion, but does not cause phase shift of the melatonin rhythm [64–66]. PACAP has also been shown to directly influence clock gene expression [67] and affect signalling pathways that integrate the molecular clock in the functionality of circadian rhythms in a dose- and phase-dependent manner [68]. Further studies are needed to determine whether the *ADCYAP1* polymorphism exerts its effect on nocturnal migratory restlessness via a phase-shift of the endogenous oscillator(s) or via a modulation of the downstream processes of the molecular clock. PACAP has also a strong modifying effect on the energy metabolism. Intracerebroventricularly administered PACAP appeared to stimulate catabolic effects on energy metabolism in chicken [69]: it increased body temperature, metabolic rate and lipid utilization. Moreover, increased PACAP concentrations in the chicken brain inhibited feeding [70], which was later shown to be mediated by corticosterone release [71]. All these shifts in metabolism and feeding behaviour have been described as the characteristics of birds preparing for or performing long-distance migratory flights [4]. Given the multiple pleiotropic functions of PACAP, *ADCYAP1* has at least the potential to modify multiple physiological and behavioural changes during the migratory period. Similar pleiotropic regulators have been postulated to explain expression covariation in large gene sets for different life-history transitions, including a migration state transition, in the Atlantic salmon [72].

Comparative studies suggest that migratory activity and/or residency have rapidly and independently evolved in different bird lineages as a response to environmental changes, and recently to global warming [21,26,73]. The existence of a strong positive correlation between the frequency of migrants in a population and the average migratory activity of individuals has led to the threshold model hypothesis [27]. This model links the continuous trait of migratory restlessness to the phenotypic dichotomy between migrants and non-migrants. We hypothesize that adaptive allele frequency shifts at the *ADCYAP1* locus could modulate population migratory activity and, as a consequence, the frequency of migratory and resident individuals in a population.
